# Attachment Networks in Committed Couples

**DOI:** 10.3389/fpsyg.2019.01105

**Published:** 2019-05-14

**Authors:** Lucia L. Carli, Elena Anzelmo, Stefania Pozzi, Judith A. Feeney, Marcello Gallucci, Alessandra Santona, Angela Tagini

**Affiliations:** ^1^Department of Psychology, University of Milan-Bicocca, Milan, Italy; ^2^School of Psychology, The University of Queensland, Brisbane, QLD, Australia

**Keywords:** attachment network, committed couples, parenting choice, primary figure, attachment functions, attachment strength, full-blown attachment

## Abstract

This study explored attachment networks in committed couples who differed in parenting choice and relationship status. Attachment networks were defined in terms of attachment functions, attachment strength, the presence of a primary figure, and full-blown attachments. Participants were 198 couples, married or cohabiting, either expecting their first child or childless-by-choice. Results indicated that participants relied most strongly on partners for all attachment functions except secure base, for which they relied on mothers to a similar extent. Furthermore, expectant women reported more proximity seeking and stronger attachments to mothers, while expectant men relied more on fathers for safe haven. Married participants indicated less proximity seeking to partners than cohabiting couples, and married women reported less reliance on partners for safe haven than married men and cohabiting women. This study supports previous findings underlining the particular importance of partners for members of committed couples. Further, it extends past research by showing the robustness of this finding across parenting choice, and by revealing gender differences in the attachment networks of committed couples.

## Introduction

Attachment theory was initially formulated to explain the bonds between children and their caregivers. However, Bowlby (1969/1982, 1973, 1980) hypothesized that attachment bonds are vital throughout the lifespan. Consequently, numerous studies have explored the nature and relevance of attachment bonds beyond childhood, and attachments to figures other than parents (e.g., Ainsworth, 1989; Weiss, 1991; Fraley and Davis, 1997; Balenzano, 2010). New attachments are created through a gradual process, during which attachment functions are attributed to individuals and attachment networks evolve (Hazan and Zeifman, 1994; Fraley and Davis, 1997; Campa et al., 2009). These networks change in relation to critical life events, such as developing a stable couple relationship and deciding to become a parent. The current study focused on the under-researched issue of attachment networks in stable couples who decide either to have children or to remain childless; gender differences were also considered.

Attachment research has highlighted both continuity and change in attachment bonds across the lifespan. Similarly to attachment patterns in childhood, studies have evidenced secure and insecure patterns in romantic partnerships (Collins and Feeney, 2000), and in relationships with friends and siblings (Trinke and Bartholomew, 1997; Umemura et al., 2014). Another similarity across the lifespan involves the critical functions of attachment bonds (Bowlby, 1969/1982; Weiss, 1982, 1991; Ainsworth, 1989); namely, the wish to establish and maintain closeness to attachment figures (proximity seeking); distress experienced in response to separation from those figures (separation protest); seeking comfort and support in stressful or unsafe conditions (safe haven); and reliance on those figures as a base from which to explore the world (secure base). These functions characterize adult couple bonds (e.g., Hazan and Shaver, 1994; Zeifman and Hazan, 1997), as well as those with other attachment figures (Trinke and Bartholomew, 1997; Doherty and Feeney, 2004; Tancredy and Fraley, 2006; Fraley and Tancredy, 2012; Umemura et al., 2014).

Despite these similarities between childhood and adult attachment, changes are also noteworthy. First, while infant attachment is based on asymmetrical roles of caregiver and careseeker, adult attachment involves reciprocal (symmetrical) relations. From adolescence onwards, individuals begin to be able to both provide and receive support from significant others, based on internalized caregiving schemata (Ainsworth, 1989; Hazan and Zeifman, 2008). Secure attachment relations with caregivers allow the development of more symmetrical relations with parents, negotiation of autonomy from the family of origin, and exploration of new symmetrical bonds outside the family (e.g., Waters and Cummings, 2000; Allen, 2008; Dinero et al., 2011).

Further, this transition from asymmetrical to symmetrical attachment bonds constitutes a crossroad for the integration of other motivational systems central to romantic relationships: the affiliative, sexual, and caregiving systems (Mikulincer and Goodman, 2006; George and Solomon, 2008; Rosenthal and Kobak, 2010). The affiliative and attachment systems partially overlap; in adolescence and adulthood, proximity-seeking behaviors can be motivated by the need for comfort or security (the attachment system), but also by a desire to engage in activities involving shared interests (Mikulincer and Selinger, 2001). The sexual system can activate proximity-seeking behaviors in romantic relationships, in turn facilitating more enduring and intimate bonds (Hazan and Diamond, 2000). Experiences of having received adequate caregiving from parents and support from a partner can then influence the decision to engage in asymmetrical caregiving of children.

In summary, new attachment figures emerge during the lifespan, and changes occur in their relative importance and the attachment functions they fulfill. Hazan and Zeifman (1994) noted that the proximity-seeking function is ‘transferred’ to peers in middle childhood, followed by safe haven and separation protest in adolescence. The secure base function is the last to be reassigned, and can still be carried out by parents during early adulthood (Fraley and Davis, 1997; Mayseless, 2004; Zhang et al., 2011). Further, the gradual affective investment in figures outside the family of origin does not involve parents being completely replaced as attachment figures; rather, they are relied on to different degrees for different functions (Trinke and Bartholomew, 1997).

The extent to which a target figure is relied upon to fulfill different attachment functions indicates the attachment strength to that individual (Doherty and Feeney, 2004; Milyavskaya and Lydon, 2012). Different attachment figures are organized in a hierarchy on the basis of attachment strength, with the primary attachment figure at the top of the hierarchy (Bretherton, 1985; Collins and Read, 1994). As the primary attachment figure may not carry out all attachment functions, the term full-blown attachment describes a figure who is relied upon for all functions (Hazan and Zeifman, 1994). The above-mentioned concepts specify the relative importance of attachment figures and describe the *attachment network.*

Research indicates that the attachment network changes across the life-cycle, tending to become more complex. *Friends* can be important for proximity seeking and safe haven but rarely become full-blown attachments, except when individuals attempt to compensate for insecure or conflictual attachments to parents (Mayseless, 2004; Nickerson and Nagle, 2005; Pitman and Scharfe, 2010; Rosenthal and Kobak, 2010). In early adulthood, the importance of friends tends to diminish, especially when individuals form committed couple relationships (Markiewicz et al., 2006; Rosenthal and Kobak, 2010; Umemura et al., 2014). Further, attachment bonds often form between *siblings*; these bonds tend to diminish in early adulthood but increase in the elderly, being stronger for individuals without a romantic partner (Doherty and Feeney, 2004; Keren and Mayseless, 2013).

Although parents continue to be relied upon as a secure base, patterns differ for mothers and fathers. Mothers tend to maintain a privileged role in the network, remaining a primary attachment figure for offspring until early adulthood (Markiewicz et al., 2006; Campa et al., 2009; Keren and Mayseless, 2013). For adults, ties to mothers can persist as full-blown attachments (Doherty and Feeney, 2004). Fathers also play an important role as secure base during adolescence and early adulthood (Umemura et al., 2014). However, they rarely constitute primary attachment figures, tending to occupy a more peripheral position in the network, especially for those in couple relationships (Trinke and Bartholomew, 1997; Markiewicz et al., 2006; Freeman and Almond, 2010). Moreover, gender differences have been found for relationships to fathers: *compared to females*, males rely to a greater extent on their fathers as safe havens (Markiewicz et al., 2006), and *also* have stronger attachments to them (Trinke and Bartholomew, 1997).

The *establishment of a couple relationship* is among the most critical events inducing changes in attachment networks. In stable couples, partners tend to develop full-blown attachments to each other, and consider them as primary attachment figures (Hazan and Zeifman, 1994; Doherty and Feeney, 2004; Feeney, 2004). The central role of romantic partners is somewhat greater for males: men rely on their partners more as a secure base, report stronger overall attachment to them, and more often consider them the primary attachment figure (Heffernan et al., 2012; Keren and Mayseless, 2013). Conversely, women report stronger attachments to parents, siblings and friends (e.g., Markiewicz et al., 2006; Umemura et al., 2014).

There is debate regarding *when* romantic partners take on their central attachment role. Some studies focus on relationship length, noting that partners become full-blown attachment figures only in committed relationships (Balenzano, 2010; Fagundes and Schindler, 2012; Heffernan et al., 2012). Others focus on the role of couple commitment and relationship status; for instance, the transition from dating to cohabiting or marriage is associated with partners’ increasing roles as attachment figures, as evidenced by scores on secure base, overall attachment strength, and reliance on partner as primary attachment figure (e.g., Feeney, 2004; Zayas et al., 2015).

The *transition to parenthood* can also modify attachment networks. The birth of a first child is marked by diminished attachment strength to partner and friends, and increased attachment to parents (Alexander et al., 2005). As Stern (1995) postulated, new mothers tend to re-evaluate their experience of their own mothers as caregivers during childhood. In societies in which the extended family still plays an important role, maternal figures often constitute the main support matrix for mothers. Further, parents of older children tend to be less attached to their own parents, shifting from their family of origin to their current family, in contrast to individuals who are childless (Doherty and Feeney, 2004).

Although research highlights the transition to parenthood as a critical event influencing the attachment network, the choice of *embarking* on parenthood should also be investigated. The last 20 years have seen an increase in the number of couples who decide to remain childless (e.g., Albertini and Mencarini, 2014). This phenomenon has been studied in relation to socio-economic status, explicit motivations, personality and gender identity (e.g., Carmichael and Whittaker, 2007; Peterson, 2014). However, research on the intergenerational determinants of this choice and its possible links with attachment bonds to the family of origin and to romantic partners is just beginning.

From a theoretical position, the choice to become a parent is associated with the development of a caregiving system, based on attachments experienced during infancy and revisited during young adulthood (George and Solomon, 2008). A transition occurs from self as careseeker to self as caretaker of another’s developmental needs (Solomon and George, 1996; George and Solomon, 2008). The choice to become a parent is consolidated by forming a stable, future-oriented relationship in which partners practice caregiving and engage in mutual emotional support. If this process does not unfold, the choice to remain intentionally childless may, in some cases, reflect a less developed caregiving ability. Studies of attachment style and parenting choice suggest that avoidant pregnant women show less interest in becoming mothers (Rholes et al., 2006; Carli et al., 2016); conversely, the desire to become a parent is greater in secure (Cheng et al., 2015) and anxious-ambivalent individuals (Carli et al., 1995). However, to date, in-depth studies on the way in which childless-by-choice individuals relate to their partners or other attachment figures have not been carried out.

As noted, much remains to be understood about the attachment networks of committed couples. Most previous studies sampled adolescents or young adults involved in relatively new romantic relationships, focusing mainly on the transfer of attachment functions from parents to partners. Moreover, studies that did investigate committed couples (Feeney and Hohaus, 2001; Doherty and Feeney, 2004; Balenzano, 2010; Keren and Mayseless, 2013), examined couples’ attachment figures at different ages and life situations (e.g., transition to parenthood), but did not specifically consider couples who were childless-by-choice.

Our study thus aimed to explore *attachment networks* in adult couples who differed in their relationship status (cohabiting or married) and parenting choice (expecting their first child or childless-by-choice), and to examine gender differences. Specifically, this study had two main objectives: (a) to investigate attachment networks in the overall sample of committed couples, and (b) to investigate differences in networks according to relationship status and parenting choice. The main effect of gender and its potential interaction with relationship status and parenting choice was also investigated, since gender differences have been previously found. Finally, possible interactions between relationship status and parenting choice were examined.

## Materials and Methods

### Participants

Participants in this study were of Italian origin and lived in northern Italy. The study was approved by the Institutional Review Board of the University of Milan-Bicocca. All participants provided written informed consent after reading a detailed description of the study. The sample consisted of 198 couples (396 individuals), divided into two groups according to their parenting choice: 94 couples were childless-by-choice, and 104 couples were expecting their first child. Childless couples were recruited by means of snowball sampling, with the following inclusion criteria: being childless by choice; not having had children from previous relationships; and for women, being of a fertile age. The average age in this group was 45.53 years (*SD* = 8.67) for men and 42.68 (*SD* = 8.59) for women. Two-thirds (*N* = 62, 65.96%) of the childless-by-choice couples were married and 34.04% (32) were cohabiting. The mean length of relationship (marriage or cohabitation, including any pre-marriage cohabitation) was 13.03 (*SD* = 8.99) years. Education level and occupation of participants in this group are reported in Table 1.

**Table 1:** Childless by choice couples – education and professional qualification.

Education	Men	Women
Degree	35.11%	44.69%
High school diploma	46.81%	45.74%
Middle school diploma	17.02%	9.57%
Elementary school diploma	1.06%	
**Professional qualification**	**Men**	**Women**
Managers	23.40%	28.72%
Technicians	9.57%	13.83%
Employees	41.49%	43.62%
Workers	10.64%	3.19%
Freelance professionals	5.32%	3.19%
Homemakers/retired	4.26%	4.26%
Unemployed	5.32%	3.19%


Couples expecting their first child were recruited during antenatal classes organized by various hospitals in the Lombardy region of northern Italy; the only inclusion criterion was expecting a first child. The average age in this group was 34.22 (*SD* = 4.33) years for men and 32.23 (*SD* = 3.71) for women. Eighty-one (77.89%) couples were married and 23 (22.11%) were cohabiting; the mean length of their cohabitation or marriage (including pre-marriage cohabitation) was 3.08 years (*SD* = 2.74). The mean length of pregnancy was 30.13 (*SD* = 6.87) weeks. Table 2 shows the educational and professional qualifications for participants in this group.

**Table 2:** Expectant couples – education and professional qualification.

Education	Men	Women
Degree	33.65%	52.89%
High school diploma	50.00%	46.15%
Middle school diploma	16.35%	0.96%
**Professional qualification**	**Men**	**Women**
Managers	15.38%	17.31%
Technicians	13.46%	17.31%
Employees	59.62%	58.66%
Workers	7.69%	2.88%
Freelance professionals	2.88%	2.88%
Homemakers/Retired		0.96%


To test for differences between expectant couples and childless-by-choice couples with regards to age and length of marriage/ cohabitation (as these may affect the results), independent sample *t*-test was used. Significant differences emerged on both these variables. Specifically, men in expectant couples were younger (*M* = 34.22, *SD* = 4.33) than men in childless couples (*M* = 45.53, *SD* = 8.67); *t*(133.61) = -11.31, *p* < 0.001, *g* = -1.67. Similarly, women in expectant couples were younger (*M* = 32.23, *SD* = 3.71) than those in childless couples (*M* = 42.68, *SD* = 8.59); *t*(123.73) = -10.91, *p* < 0.001, *g* = -1.60. Further, childless couples had longer relationships (cohabitation or marriage) (*M* = 13.03, *SD* = 8.99) than expectant couples (*M* = 3.08, *SD* = 2.74); *t*(221.89) = -14.57, *p* < 0.001, *g* = -1.75. Participants who were expecting their first child did not differ from childless-by-choice participants in terms of relationship status (being married or cohabiting) (χ^2^ = ns).

### Procedure

Participants were given a short description of the study aims and provided informed consent. Each couple member was asked to indicate socio-demographic data, relationship and parenting status and length of marriage/cohabitation, and to complete a questionnaire investigating their attachment network. A research assistant briefly explained the content and meaning of the study before participants began completing the questionnaires. The assistant was available for any questions during the 30 min needed for the questionnaire. All the questionnaires were anonymous and participants could withdraw from the study at any time. To ensure confidentiality, the questionnaire was given to each individual, who was instructed to place it in an envelope once completed. The envelope was then collected by the researchers or by acquaintances through whom the couple had been contacted. This study was approved by the Institutional Review Board of the University of Milan-Bicocca.

### Measures

Participants completed a n Italian self-report version of the WHO-TO scale, similar to that adopted by Doherty and Feeney (2004). Standard steps guided the translation process used in this study. First, three independent researchers translated the questionnaire from English to Italian and then reached agreement on a common version. The shared form was then back-translated by a bilingual individual with extensive knowledge of psychological research. Originally developed by Hazan and Zeifman (1994), the WHO-TO is a 12-item interview comprising four three-item subscales, one for each attachment function. The instrument asked respondents to name the single figure they would turn to for Proximity Seeking, Secure Base, Safe Haven, and Separation Protest. Whereas Doherty and Feeney employed only eight items, the instrument in the current study contained all 12 items of the original interview, but in self-report format. Moreover, rather than asking respondents to name a single person for each item, we (like Doherty and Feeney) asked them to list up to five attachment figures, and to rank them in order of importance. This adaptation of the original scale integrated the items of the WHO-TO with the response format of the Attachment Network Questionnaire (Trinke and Bartholomew, 1997), in order to explore attachment networks more fully.

The scoring procedure we used mirrored that of Doherty and Feeney. Specifically, each attachment figure listed was assigned a score from five (for the first figure named) to one (for the last figure named). By adding the scores obtained by each figure across the three items for each attachment function, an index (ranging from 1 to 15) was computed, indicating the *reliance on that figure for that function*. Higher scores imply greater reliance on a figure. If participants listed the same kind of attachment figure more than once within a single item (e.g., different friends or siblings), only the first one was assigned a score.

To capture the relative importance of each figure within the network, three indexes were calculated. First, *attachment strength* (ranging from 1 to 15) was the mean score obtained by each figure across the four attachment functions; reliability for the 12-item scale was good, with alpha coefficient ranging from 0.92 for fathers to 0.84 for relatives. Second, *full-blown attachment* was defined by a figure scoring eight or higher on each of the four functions, thus obtaining a total score of 32 or higher. (Doherty and Feeney, 2004 defined full-blown attachment by high scores on three functions only, omitting proximity seeking since this behavior may reflect various motivations; the current criterion provides a comprehensive but stringent test of full-blown attachments.) Finally, a *primary attachment* was the figure with the highest score across the four functions (this need not necessarily constitute a full-blown attachment). Full-blown attachment and primary attachment figures were turned into dichotomous variables, with 1 indicating that the figure met the particular criteria, and 0 indicating that he/she did not.

### Data Analysis

#### Overall Attachment Networks

To gain an overview of participants’ attachment networks, we examined the responses given by participants in both samples and computed descriptive statistics for reliance on attachment figures and attachment strength to the figures listed for each attachment function. Frequencies for full-blown and primary attachment figures were also computed. To further explore differences between the various figures with regards to attachment functions and attachment strength, a set of Repeated-Measures ANOVAs with Huynh–Feldt correction was run on the full sample (irrespective of parenting choice).

#### Comparing the Samples

Differences in attachment networks. Differences in attachment networks in relation to gender, relationship status (marriage vs. cohabitation) and parenting choice (expecting vs. childless couples) were explored using mixed-effects modeling. Couple members were included as clusters in the model due to the observed interdependence between their scores, as computed by the intraclass correlation coefficient. Gender, relationship status, parenting choice and their interactions were included in the model as fixed effects, and their influence was tested on the continuous dependent variables (reliance on target figure for each attachment function and attachment strength). Given differences between the two samples in terms of age and length of marriage/cohabitation, these variables were entered in the model as covariates.

To determine the effects of gender, parenting choice and relationship status on full-blown attachment and primary attachment, a set of hierarchical binary logistic regressions was run. Age and length of marriage/cohabitation were entered as control variables in the first block; the second block contained the independent variables. Only participants who had a primary attachment figure (*N* = 381) were included in the analyses for that variable.

## Results

### Aim 1: Overall Attachment Networks

Seventeen categories of target figures fulfilling attachment functions were identified. In order to minimize dispersion, categories referring to family members other than parents and siblings were grouped and recoded as ‘relatives.’ Thus, six target figure categories (partners, mothers, fathers, siblings, friends, and relatives) were employed in all subsequent analyses.

Descriptive statistics for *reliance* and *attachment strength* to each target figure were computed for each attachment function, as indicated in Table 3. Partners were preferred over all other figures for the four attachment functions, and hence showed the highest attachment strength.

**Table 3:** Reliance on targets for each attachment function and attachment strength (*N* = 396).

Target figures	Proximity seeking		Separation protest		Safe haven		Secure base		Attachment strength	
	*M*	*SD*	*M*	*SD*	*M*	*SD*	*M*	*SD*	*M*	*SD*
Partner	13.09	3.91	12.43	4.66	12.12	4.75	10.59	5.50	12.06	3.60
Mother	1.13	2.68	2.20	3.83	3.33	4.59	4.44	4.62	2.77	2.97
Father	0.36	1.38	0.88	1.19	3.16	2.86	4.80	2.86	1.32	2.38
Sibling	1.12	2.90	0.53	1.87	1.57	3.54	1.52	3.60	1.18	2.35
Friend	6.82	4.86	1.09	2.86	4.06	4.77	0.55	1.78	3.13	2.64
Relatives	0.72	2.37	0.68	2.41	0.73	2.57	0.85	2.76	0.75	2.01


The importance of partners in committed couples’ attachment network is confirmed by the *full-blown attachment* data: partners constituted full-blown attachments for 59.09% of the sample (*N* = 234), whereas other figures met this criterion in very small percentages (ranging from 0 to 1.6%). When *primary attachment figures* were considered, 15 participants (3.79%) were found to have more than one primary attachment; of the remaining 381 participants, 353 chose partners (92.65%), with small percentages for other figures (from 5.3% for friends to 0.3% for relatives).

Repeated-measures ANOVAs showed significant differences between the six attachment figures in terms of being relied on for all four attachment functions (see Table 3 for mean scores, and Table 4 for details of significance tests). *Post hoc* tests with Bonferroni correction showed that partners were relied on more than all other targets for all four functions (all *p* < 0.001). For *Proximity Seeking*, partners were followed by friends, who were relied on more than mothers, fathers, siblings, and other relatives (all *p* < 0.001). Regarding *Separation Protest*, partners were followed by mothers, who scored higher than fathers, siblings, friends, and relatives (all *p* < 0.001). For *Safe Haven*, friends and mothers were relied on more than all other targets (except partners) (all *p* < 0.001), and siblings were relied on more than relatives (*p* < 0.01). Finally, for *Secure Base*, partners and mothers were relied upon more than other figures (fathers, siblings, friends, relatives) (all *p* < 0.001).

**Table 4:** Results of repeated measures ANOVAs.

	*F*	*df1*	*df2*	*p*	η*^2^*
Proximity seeking	922.45	3.30	1305.30	<0.001	0.70
Separation protest	860.45	3.79	1496.03	<0.001	0.68
Safe haven	8888.90	4.05	1601.52	<0.001	0.51
Secure base	332.66	3.99	1577.96	<0.001	0.46


There were also significant differences between figures in *attachment strength*, *F*(4.53,1788.45) = 944.50, *p* < 0.001, η^2^ = 0.70. *Post hoc* tests with Bonferroni correction showed stronger attachment to partners (*M* = 12.06, *SD* = 3.60), than to all other figures (father, *M* = 1.32, *SD* = 2.38, mother, *M* = 2.77, *SD* = 2.97, siblings, *M* = 1.18, *SD* = 2.35, relatives, *M* = 0.75, *SD* = 2.01, friends, *M* = 3.13, *SD* = 2.64), all *p* < 0.001. Partners were followed by friends, to whom participants reported stronger attachment than to fathers (*p* < 0.001), siblings (*p* < 0.001), and relatives (*p* < 0.001), but not to mothers (*p* = 1.00).

### Aim 2: Networks as a Function of Relationship Status, Parenting Choice, and Gender

Regarding the degree of *reliance on targets* for the four attachment functions, mixed-effects models revealed significant main and interactive effects of relationship status, parenting choice and gender. For *Proximity Seeking*, relationship status showed a main effect for partners only *F*(1,194.52) = 8.41, *p* < 0.01: cohabiting couples reported seeking more proximity to partners (*M* = 14.32, *SD =* 2.54) than married couples (*M =* 12.61, *SD =* 4.23). Relationship status interacted with parenting choice for proximity seeking to fathers, *F*(1,192.24) = 8.33, *p* < 0.01; cohabiting expectant couples reported seeking more proximity to their fathers (*M* = 0.78, *SD =* 1.60) than cohabiting childless couples (*M* = 0.00; *SD* = 0.00), *p* *<* 0.01. Further, a significant main effect of gender emerged for *Proximity Seeking* to siblings, *F*(1,217.47) = 9.42, *p* < 0.01, friends*, F*(1,218.71) = 6.90, *p* < 0.01, and mothers, *F*(1,218.45) = 11.82, *p* < 0.01. Women sought more proximity to siblings (*M =* 1.69, *SD =* 3.58) than did men (*M* = 0.55, *SD =* 1.84), while men sought more proximity to friends (*M =* 7.57, *SD =* 4.72) than women (*M =* 6.08, *SD =* 4.90). For Proximity Seeking to mothers, the main effect of gender was qualified by an interaction between gender and parenting choice, *F*(1,194.61) = 6.59, *p* < 0.05. *Post hoc* analyses revealed that women expecting their first child sought more proximity to their mothers (*M =* 2.30, *SD* = 3.71) than did expectant men (*M =* 0.61, *SD =* 1.46), childless men (*M* = 0.55, *SD =* 1.82) and childless women (*M* = 0.98, *SD =* 2.73) (all *p* < 0.001).

*Separation Protest* from fathers revealed a significant interaction between relationship status and parenting choice, *F*(1,192.39) = 4.89, *p* < 0.05. *Post hoc* tests showed that cohabiting childless couples reported less Separation Protest from fathers (*M* = 0.27, *SD =* 1.31) than expectant couples, both married (*M* = 1.50, *SD =* 2.64) and cohabiting (*M* = 0.99, *SD =* 3.24), all *p* < 0.05. Significant main effects of gender emerged for mothers, *F*(1,221.82) = 7.42, *p* < 0.01, and siblings, *F*(1,217.86) = 8.48, *p* < 0.01. Women protested separation from their mothers (*M =* 2.64, *SD =* 4.09) and siblings (*M* = 0.89, *SD* = 2.34) to a greater extent than men (respectively, *M* = 1.76, *SD =* 3.51 and *M* = 0.16, *SD =* 0.89).

For *Safe Haven*, significant main effects of gender emerged for reliance on mothers*, F*(1,221.84) = 9.30, *p* < 0.01, and siblings, *F*(1,217.48) = 5.94, *p* < 0.05. Women relied on mothers (*M =* 4.04, *SD* = 4.87) and siblings (*M =* 2.09, *SD* = 4.02) more than men did (respectively, *M =* 2.62, *SD =* 4.17, and *M =* 1.04; *SD =* 2.88). A significant interaction between gender and relationship status emerged for reliance on partners as a Safe Haven, *F*(1,195.04) = 9.70, *p* < 0.01. *Post hoc* tests revealed that married women reported less reliance on partners for Safe Haven (*M =* 10.97, *SD =* 5.37) than cohabiting women (*M =* 13.09, *SD =* 3.66), *p* < 0.01 and married men (*M =* 12.88, *SD =* 4.27), *p* < 0.001. A significant interaction between gender and parenting choice for reliance on fathers as Safe Haven, *F*(1,195.60) = 7.73, *p <* 0.01, showed that expectant men relied on fathers (*M =* 2.24, *SD =* 4.04) more than expectant women (*M* = 0.62, *SD =* 2.40), childless men (*M* = 0.85, *SD =* 2.48), and childless women (*M* = 0.99, *SD* = 3.16), all *p* < 0.01.

Lastly, for *Secure Base*, significant main effects of gender emerged for mothers, *F*(1,221.12) = 5.35, *p* < 0.05, and partners, *F*(1,222.08) = 15.17, *p* < 0.001. Women reported more reliance on mothers as a Secure Base (*M =* 5.10, *SD* = 4.65) than did men (*M =* 3.77, *SD* = 4.51), while men reported more reliance on partners (*M =* 11.66, *SD* = 4.76) than did women (*M =* 9.52, *SD* = 5.97). A significant interaction between gender and relationship status for reliance on siblings, *F*(1,195.13) = 5.59, *p <* 0.05, showed that reliance on siblings as a Secure Base was greater for cohabiting men (*M =* 2.29, *SD* = 4.42) than married men (*M* = 1.08, *SD* = 2.87), *p* < 0.05.

Overall *attachment strength* revealed a significant interaction between relationship status and parenting choice, *F*(1,192.35) = 4.27, *p* < 0.05, for attachment to fathers: cohabiting expectant couples had stronger attachment to fathers (*M* = 2.11, *SD* = 2.98) than childless couples, both cohabiting (*M* = 0.88, *SD* = 1.62) and married (*M =* 1.04, *SD* = 2.38), all *p* < 0.01. There were also significant main and interaction effects of gender and parenting choice for attachment to mothers, siblings and fathers. Specifically, a main effect of gender, *F*(1,221.80) = 14.53, *p* < 0.001, as well as an interaction between gender and parenting choice, *F*(1,195.75) = 4.75, *p* < 0.05, emerged for attachment to mothers. Expectant women had stronger attachment to mothers (*M* = 4.05, *SD* = 3.27) than expectant men (*M* = 2.38, *SD* = 2.51), childless women (*M* = 2.60, *SD* = 2.94) and childless men (*M* = 1.96, *SD* = 2.73), all *p* < 0.001. For attachment strength to siblings, there was a significant main effect of gender *F*(1,218.34) = 6.16, *p* < 0.05, and a significant interaction between gender and relationship status, *F*(1,195.08) = 4.62, *p* < 0.05. *Post hoc* analyses revealed that married women reported stronger attachment to their siblings (*M* = 1.74, *SD =* 2.99) than married men did (*M* = 0.66, *SD* = 1.66) (*p* < 0.001).

Hierarchical binary logistic regressions examining *full-blown attachment* showed significant effects only with regards to partner. The final model containing all predictors (control variables, plus gender, parenting choice, and relationship status) was significant, indicating that it could distinguish between subjects who had chosen their partner as the full-blown attachment figure and those who had not χ^2^(5,396) = 13.09, *p* = 0.02, explaining 4.4% of the variance (Cox & Snell, R Square). However, only a main effect for gender (Wald = 6.23, *p* = 0.01) was observed; Men were more likely (64.6 vs. 53% OR 1.59, 95%) to choose their partner as full-blown attachment figure. Similar hierarchical regressions conducted on the subsample of people who reported having only one *primary attachment figure* (*N* = 381) revealed no significant main or interaction effects of the variables (control or independent) on the choice of figure.

## Discussion

This study extends our understanding of the attachment networks of committed couples. In line with previous studies, multiple target figures were identified for each attachment function, and the relative position of figures in the network was described, operationalized in terms of *reliance on the target for each attachment function* (Proximity Seeking, Safe Haven, Separation Protest, Secure Base), *overall attachment strength*, presence/absence of a *full-blown attachment*, and presence/absence of a *primary attachment figure*. Extending past research, we assessed differences in attachment networks in relation to gender, relationship status, and parenting choice.

### Attachment Network Structure in Committed Couples

Consistent with previous studies (e.g., Hazan and Zeifman, 1994; Doherty and Feeney, 2004), our results clearly indicate that partners constitute the primary attachment figure for committed couples in our sample. Partners were the preferred target for all attachment functions, and as a consequence, scores for attachment strength were highest toward partners. Further, almost 60% of the individuals in our sample indicated partners as their full-blown attachment figure. These results support studies that highlight the unique role of romantic partners in adults’ attachment networks (Doherty and Feeney, 2004; Feeney, 2004; Keren and Mayseless, 2013). In our study, reliance on partners was highest for Proximity Seeking, and lowest for Secure Base. This result may reflect affiliative and sexual systems partially overlapping with the attachment system, leading adults to seek proximity to partners in order to share interests or to satisfy sexual needs, as well as for comfort or security (Hazan and Diamond, 2000; Mikulincer and Goodman, 2006).

In the present study, mothers were second only to partners for Separation Protest, Safe Haven and Secure Base functions, supporting the claim that they continue to hold a privileged role in the lives of their adult children (Doherty and Feeney, 2004; Campa et al., 2009; Keren and Mayseless, 2013). *Fathers* were third in the attachment network, but only as secure base. This result suggests that attachment to fathers is more significant for exploratory behavior than for reassurance in stressful situations (Grossmann et al., 2002; Di Folco and Zavattini, 2014; Palm, 2014). Overall, these findings suggest that although parents may lose their privileged position in the attachment network as their children mature and form romantic relationships, they continue to fulfill important attachment functions (Scharfe et al., 2017).

Siblings were third or fourth for all attachment functions, supporting previous findings suggesting that siblings are moderately important as attachment figures for adults in stable relationships (Doherty and Feeney, 2004; Schwarz et al., 2015). Friends occupied second position in the attachment network for Proximity Seeking and Safe Haven; further, in terms of attachment strength, friends emerged as the second most important figure. However, the absence of a secure base function in friends suggests that rather than the attachment system, an affiliative motivational system may be involved, implying reliance on friends for shared interests and activities (Pitman and Scharfe, 2010; Rosenthal and Kobak, 2010). Finally, relatives were the most marginal attachment figures, obtaining the lowest scores on all attachment functions.

The secondary role of target figures other than partners was confirmed when global indicators of attachment (attachment strength, full-blown and primary attachment figure) were considered. These results further attest to the preeminence of partners as attachment figures for committed couples.

### Attachment Network as a Function of Relationship Status, Parenting Choice, and Gender

In general, the relative importance of partners within the attachment network was not affected by relationship status (married or cohabiting), although differences were found for two specific attachment functions: Proximity Seeking and Safe Haven. The only main effect of relationship status concerned Proximity Seeking to partners, which was greater in cohabiting couples than in married ones. In part, this result may be due to cohabiting couples being less certain about the stability of their relationship, thus showing higher anxiety and proximity-seeking behaviors (Stanley et al., 2006). The greater vulnerability of cohabiting couples is further documented by studies underlining their susceptibility to break-ups, and their less effective communication strategies when compared to married couples (Kamp Dush et al., 2003; Kline et al., 2004). Further, this ‘cohabitation effect’ may indicate that these couples are also less likely to evaluate and reflect upon their relationship choice (Stanley et al., 2006). These results also provide indirect support for the claim that Proximity Seeking is not necessarily a strong indicator of attachment strength, but rather, may reflect the activation of other motivational systems such as the affiliative and sexual systems (Trinke and Bartholomew, 1997).

Regarding Safe Haven, an interaction between relationship status and gender emerged, with married women relying on partners to a lesser degree than cohabiting women and married men. This result may reflect traditional expectations of marital roles, with wives being expected to act as physical and emotional safe havens, and to take care of their children and husbands, rather than being recipients of these functions. Such traditional expectations may also be more evident in Italy than in some other countries. Multi-cultural studies are needed in order to test this interpretation.

Partners’ central role within the attachment network was largely independent of participants’ parenting choice (expecting or childless). For reliance on figures other than partners, parenting choice was related only to attachment to parents. Specifically, expectant women sought more proximity and were more strongly attached to their mothers than expectant men and childless-by-choice individuals. Further, expectant men relied on their fathers as safe havens more than expectant women and childless-by-choice participants. These results support previous findings (e.g., Alexander et al., 2005; Roy et al., 2014), that expectant individuals tend to seek more support and advice from their parents. Our findings also highlight the importance of mother-daughter and father-son bonds in adulthood. The former pattern supports Stern’s (1995) theory of a motherhood constellation, in which maternal figures play a fundamental role in influencing new mothers. Our results further suggest that prospective fathers may tend to develop a ‘fatherhood constellation’ (Cupa and Riazuelo-Deschamps, 2001), turning to their own fathers for support and advice. In contrast, childless couples seemed to show less reliance on parents (especially fathers). Although participants’ age was controlled for in the analyses, this finding may reflect, in part, the fact that the parents of the childless participants were somewhat older. Hence, these elderly parents may have been less available to their offspring; in some families, caregiving roles may even be reversed (or some parents may be deceased). Such changes in attachment and caregiving patterns require further cross-generational research.

We also found that expectant cohabiting couples reported more proximity seeking and separation protest in relation to fathers than cohabiting childless-by-choice individuals. Expectant cohabiting individuals also had stronger overall attachment to fathers than both cohabiting and married childless-by-choice individuals. In traditional Italian families, a rapprochement to parents during pregnancy and the birth of the first child is common and may account for these findings. However, it is worth noting the paucity of main effects of both relationship status and parenting choice. In fact, these variables were more likely to interact (with each other, and with gender) than to show main effects, despite the fact that there is greater statistical power to detect main effects than interactions. This suggests that these demographic and relational variables relate to attachment network structure through relatively complex patterns.

The present study supports previous findings (e.g., Heffernan et al., 2012; Keren and Mayseless, 2013; Umemura et al., 2014), regarding the influence of gender on attachment network structure. We similarly found that men relied more on their partners as attachment figures, particularly for the secure base function. Moreover, men tended to select their partners as full-blown and primary attachment figures to a greater extent.

Gender differences were also found for other targets. Women tended to be more reliant on mothers and siblings for all attachment functions. Further, women were more strongly attached to these figures (overall) than men, and more likely to report them as primary attachment figures. This finding fits with studies (Bombi et al., 2011), underlining the greater involvement of women in caregiving and family relationships in Italy. This result is also partially in line with Doherty and Feeney (2004), who found that women generally relied more on target figures other than partners, although, unlike in the present study, not specifically on their family of origin. Moreover, we found that men tended to seek proximity to friends to a greater degree than women, suggesting that affiliative bonds may be more relevant for males (Emde and Harmon, 1982; Moen, 1996). It is unclear if these results reflect the particular family structure in Italy, in which traditional gender roles may persist (Bimbi, 1991); further research is necessary in this respect.

### Limitations and Future Directions

This study extends our understanding of the attachment networks of committed couples. Specifically, it has identified multiple target figures and described their relative position within the networks. Further, although relationship status and parenting choice had relatively few effects on the networks, some complex effects emerged; several gender differences were also obtained. Such findings are relevant to practitioners who seek to evaluate and strengthen the sources of comfort and security available to individuals and couples, especially during important transitions.

In terms of limitations, we have already noted some features of the more traditional family structure in Italy, which may apply less in other cultures. Further, the study focused on attachment strength, rather than on the *quality* of the attachments to various figures. These two constructs are conceptually distinct, but research is needed to examine ways in which they may interact. Attachment bonds may be strong but insecure. Further, attachment bonds to peers may represent either a further development of a secure attachment to parents, or ‘compensate’ for an insecure attachment to parents (e.g., Pitman and Scharfe, 2010; Rosenthal and Kobak, 2010). Finally, because the study was cross-sectional, caution must be exercised in drawing causal inferences. This is particularly relevant to understanding how couples develop more committed relationships, and the effects of parenting choice. Expectant couples in this study were well into their pregnancy when recruited. Hence, it is possible that the high level of protest of separation from partners, evident among these couples, reflected a need for mutual support as childbirth approaches. Longitudinal and cross-generational studies are needed to assess attachment networks, both before and after critical events.

## Ethics Statement

This study was carried out in accordance with the recommendations of ‘name of guidelines,’ the Ethics Committee of the Psychology Department of the Milano-Bicocca University with written informed consent from all subjects. All subjects gave written informed consent in accordance with the Declaration of Helsinki. The protocol was approved by the Ethics Committee of the Psychology Department of the Milano-Bicocca University.

## Author Contributions

LC: conception of research question, design of study, and supervision of all phases of the research. EA: main data collection and statistical analysis. SP: further data collection and statistical analysis. JF: supervision and contribution to manuscript-writing. MG: supervision of statistical analysis. AS: supervision of data collection. AT: supervision of data analysis, interpretation and discussion of data, and main contributor to the conception and writing of manuscript.

## Conflict of Interest Statement

The authors declare that the research was conducted in the absence of any commercial or financial relationships that could be construed as a potential conflict of interest.
